# Unraveling the (De)sodiation Mechanisms of BiFeO_3_ at a High Rate with *Operando* XRD

**DOI:** 10.1021/acsami.3c17296

**Published:** 2024-02-27

**Authors:** Anders Brennhagen, Casper Skautvedt, Carmen Cavallo, David S. Wragg, Alexey Y. Koposov, Anja O. Sjåstad, Helmer Fjellvåg

**Affiliations:** † Centre for Materials Science and Nanotechnology, Department of Chemistry, University of Oslo, PO Box 1033, Blindern, N-0315 Oslo, Norway; ‡ CENATE, Centrifugal Nanotechnology, Rakkestadveien 1, 1814 Askim, Norway; § Department of Battery Technology, Institute for Energy Technology (IFE), Instituttveien 18, 2007 Kjeller, Norway

**Keywords:** BiFeO_3_, Na-ion battery, *operando* XRD, high rate cycling, conversion-alloying materials

## Abstract

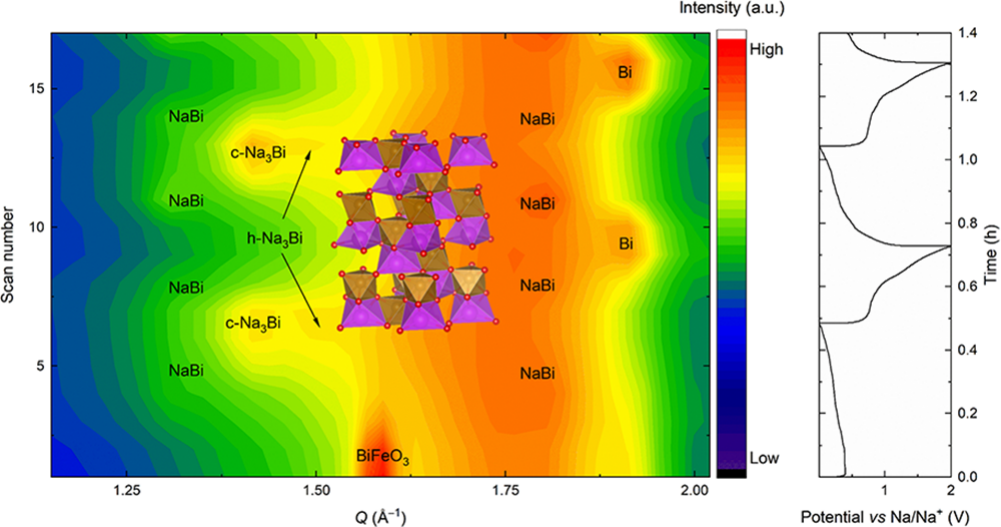

Development of new anode materials for Na-ion batteries strongly depends on a detailed understanding of their cycling mechanism. Due to instrumental limitations, the majority of mechanistic studies focus on *operando* materials’ characterization at low cycling rates. In this work, we evaluate and compare the (de)sodiation mechanisms of BiFeO_3_ in Na-ion batteries at different current densities using *operando* X-ray diffraction (XRD) and *ex situ* X-ray absorption spectroscopy (XAS). BiFeO_3_ is a conversion-alloying anode material with a high initial sodiation capacity of ∼600 mAh g^–1^, when cycled at 0.1 A g^–1^. It does not change its performance or cycling mechanism, except for minor losses in capacity, when the current density is increased to 1 A g^–1^. In addition, *operando* XRD characterization carried out over multiple cycles shows that the Bi ⇋ NaBi (de)alloying reaction and the oxidation of Bi at the interface with the Na–Fe–O matrix are detrimental for cycling stability. The isolated NaBi ⇋ Na_3_Bi reaction is less damaging to the cycling stability of the material.

## Introduction

Na-ion batteries (NIBs) have been long viewed as promising alternatives to modern Li-ion batteries (LIBs) due to the high abundance and low cost of Na that can make up for the slightly lower energy densities.^1−3^ The application of NIBs is particularly promising in stationary energy storage, where high power is necessary and energy density is less crucial.^4^ However, further optimization of NIBs for high power applications requires development of suitable anode materials, whose performance will not suffer at elevated cycling rates. High rate capabilities and ability to achieve a good compromise between high capacity and cycling stability make conversion-alloying materials (CAMs) a promising group of compounds.^5−7^ However, their complex cycling and degradation mechanisms, often involving the formation of amorphous phases, make them challenging to develop further. In order to determine the (de)sodiation mechanisms of these materials, it is essential to use *operando* methods, where the structural characterization is performed during electrochemical cycling. This methodology is superior to ex situ characterization due to potential structural relaxation of active materials and risk of side reactions with the environment when the materials are extracted from coin cells during the preparation of ex situ samples.^8−10^

The general cycling mechanism of CAMs at low cycling rates (<150 mA g^–1^) has been revealed through several studies on LIBs and NIBs with *operando* techniques including X-ray diffraction (XRD), X-ray absorption spectroscopy (XAS), and total scattering computed tomography (TSCT) coupled with ex situ and in situ transmission electron microscopy (TEM).^11−19^ These studies have confirmed a conversion reaction during the first sodiation/lithiation, which leads to a phase separation with formation of nanosized particles of the alloying element (Si, Ge, Sn, Sb, Bi) distributed in a Na_*x*_X (X = O, S, Se, Te, P, oxometallates) matrix.^6^ During the following cycles, the particles of the alloying element are the main contributors to the capacity. However, there are reports of partially reversible conversion reactions in the binary CAMs^11,18^ and electrochemical activity in the transition metal in the matrix of ternary CAMs adding to the capacity.^13,20,21^

Bi metalates are a group of ternary CAMs, which exhibit high capacities as anode materials in NIBs. BiFeO_3_, Bi_2_(MoO_4_)_3_, BiVO_4_, and Bi_2_MoO_6_ have previously been explored with primary focus on cycling stability.^14,20,22,23^ In line with the general cycling mechanism of CAMs, Bi metalates undergo an irreversible conversion reaction during the first sodiation to produce Bi nanoparticles embedded in a Na–TM–O (TM = transition metal) matrix. The Bi particles then reversibly transform into Na_3_Bi (via NaBi as an intermediate phase) during sodiation.^20,22^ There are two known polymorphs of Na_3_Bi, hexagonal (h-Na_3_Bi) and cubic (c-Na_3_Bi). The h-Na_3_Bi phase is thermodynamically stable and has been observed to form from microcrystalline NaBi particles, while c-Na_3_Bi forms in nanocrystalline NaBi particles (for example, those formed in Bi metallates).^20,24^ The buildup of h-Na_3_Bi during prolonged cycling of Bi metallates has been linked to particle growth and capacity degradation.^22^ Despite significant efforts, we still lack a generalized understanding of the evolution of Na_*x*_Bi particles and the Na–TM–O matrix during cycling.^14,20,21^

BiFeO_3_ is perhaps the most straightforward chemical system among Bi metallates. It has shown reversible (de)sodiation capacities up to 600 mAh g^–1^, but its cycling stability is limited and the operating mechanism is still disputed.^14,25^ Surendran et al. performed *operando* XAS on BiFeO_3_ and proposed the formation of metallic Fe in addition to Na_2_O and an Fe-based oxide during the initial conversion reaction.^14^ The same study also showed the presence of Bi–O bonds after the first desodiation, indicating a partial oxidation of Bi metal as was determined by Fourier transformed (FT) extended X-ray absorption fine structure (EXAFS).^14^

The majority of *operando* studies of CAMs have been performed at low current densities and, to the best of our knowledge, there are no *operando* studies addressing the cycling mechanisms of these materials at high cycling rates. This lack of insight is due to the challenges associated with conducting *operando* measurements at high rates: performing reliable electrochemistry in an *operando* cell with an electrode thick enough to obtain a sufficient intensity of X-ray signals. Another limiting factor is the availability of X-ray sources, as synchrotron radiation is needed to obtain high-quality data with high time resolution. In this work, we conducted *operando* XRD measurements, with current densities of 0.1 and 1 A g^–1^, to elucidate and compare the electrochemical cycling mechanism of BiFeO_3_ at low and high rates. We also deployed *operando* XRD over 27 (de)sodiation cycles combined with ex situ XAS to gain a further understanding of the interplay between the Na_*x*_Bi alloying particles and the Na–Fe–O matrix and their influence on the cycling stability of BiFeO_3_.

## Results and Discussion

### Material and Electrochemical Characterization

Synthesis of phase pure BiFeO_3_ is recognized as challenging owing to competing ternary phases.^25^ After optimization of the reaction conditions, we managed to synthesize BiFeO_3_ with small amounts of Bi_2_O_3_ (∼12 wt % according to Rietveld refinement) by means of a sol–gel synthetic route (Figure S1, Section S1, SI). The Bi_2_O_3_ impurities were successfully removed by leaching with nitric acid (Figure S2, SI), but the resulting product showed poor electrochemical performance (Figure S3, SI). Therefore, the unleached product was used for further work.

The electrochemical performance of BiFeO_3_ was evaluated at different cycling rates with both galvanostatic cycling (GC) and cyclic voltammetry (CV) (Figure S4, Section S2, SI). The initial sodiation capacity of BiFeO_3_, obtained from GC measurements, was ∼600 mAh g^–1^ at 0.1 A g^–1^, comparable to that of literature reports.^14,25^ In addition, fast cycling was undertaken at 1 A g^–1^ where BiFeO_3_ maintained a reasonable performance. The cycling stability, even at 0.1 A g^–1^, is nevertheless challenging, and the capacity drops from ∼450 mAh g^–1^ to ∼130 mAh g^–1^ after 25 cycles.

### Elucidation of the Cycling Mechanism at Low and High Rates

**Figure 1:**
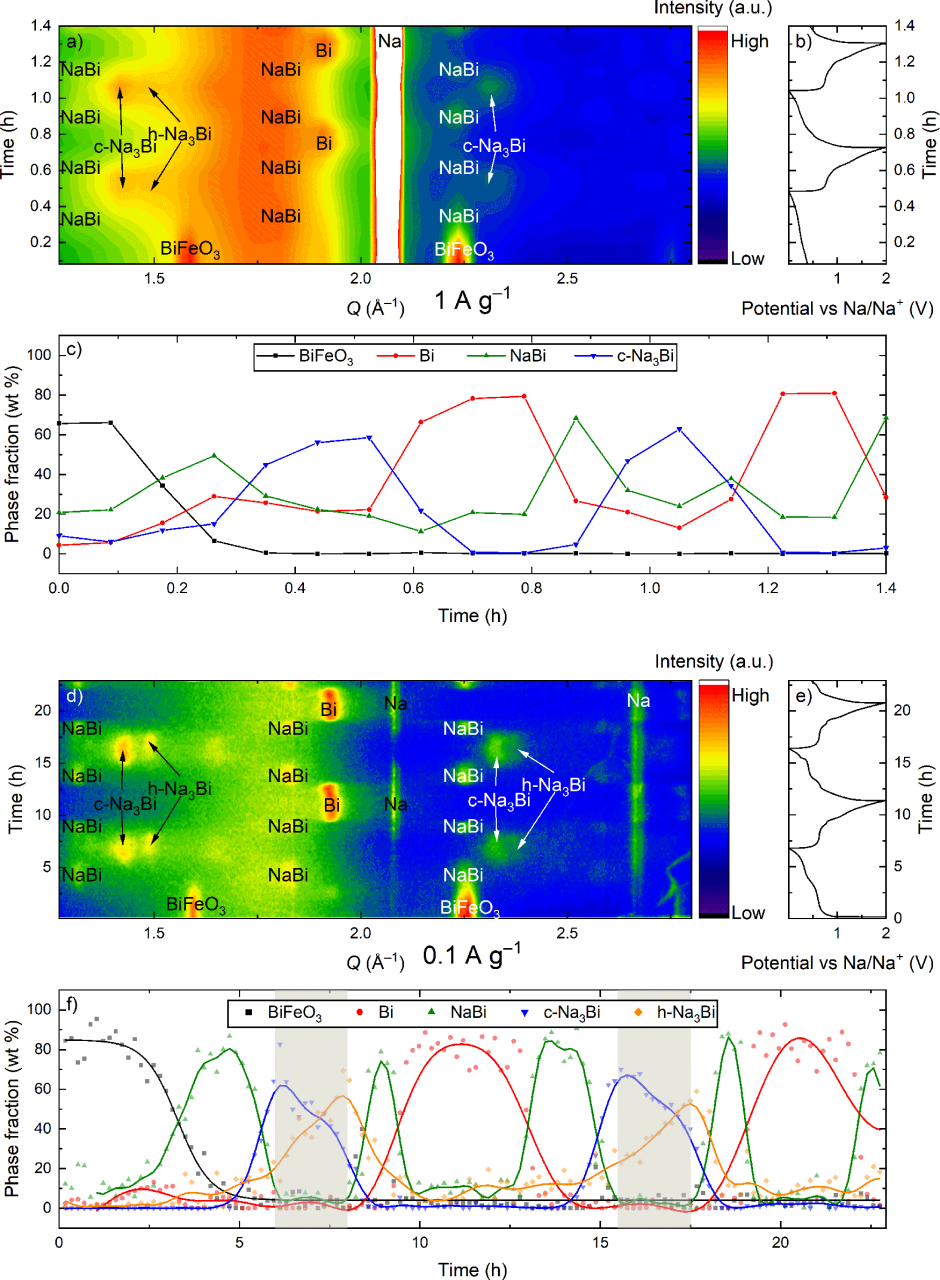
(a) Contour plot derived from the high-rate *operando* XRD measurement of 2 (de)sodiation cycles of BiFeO_3_, measured with synchrotron radiation at BM31 at ESRF and (b) corresponding (de)sodiation curves measured at 1 A g^–1^. (c) Phase fractions in wt % retrieved from the surface Rietveld refinement of the diffraction patterns plotted in (a), showing the evolution of the Bi-containing phases as a function of time. The h-Na_3_Bi phase is omitted from the surface Rietveld refinement due to limited data quality at a high data collection rate. (d–f) Corresponding graphs for *operando* XRD measurement from the home lab with a current density of 0.1 A g^–1^. The gray areas in (f) highlight the regions where only c-Na_3_Bi and h-Na_3_Bi are present.

Due to experimental limitations, *operando* studies are typically performed under slow cycling conditions, often quite different from the cycling protocols to which electrode materials are subjected in normal use. *Operando* characterization at high cycling rates is rare, despite the fact that understanding of the material’s behavior under such conditions is critical for the development of batteries with high power.^26,27^ Several CAMs have shown promising electrochemical performance at high rates,^25,28,29^ but their cycling mechanisms at these high current densities have not been studied with *operando* methods. To address this problem, we performed *operando* XRD on BiFeO_3_-based electrodes using synchrotron radiation at BM31 at the European Synchrotron Radiation Facility (ESRF) during electrochemical cycling at 1 A g^–1^ in a half-cell configuration using Na foil as a counter electrode (Figure 1a–c). Surprisingly, BiFeO_3_ is fully sodiated to Na_3_Bi in the first sodiation despite the high current density, confirming that BiFeO_3_ can maintain the same cycling mechanism and capacity at 1 A g^–1^ as at lower cycling rates.^14,25^ Overall, the cycling mechanism was found to be similar to that proposed previously for BiFeO_3_ and confirmed by *operando* and *post-mortem* studies in other Bi metallates.^14,20,22,25^ The minor, but significant difference for BiFeO_3_ compared to other Bi metallates is the formation of both h-Na_3_Bi and c-Na_3_Bi phases during the first sodiation, regardless of the current density. This is different from what was shown for BiVO_4_, Bi_2_(MoO_4_)_3_, and Bi_2_MoO_6_ as they formed only c-Na_3_Bi during the first cycles.^20,22^ The formation of h-Na_3_Bi has been shown to form in microsized NaBi particles, while c-Na_3_Bi forms from nanosized NaBi particles.^24^ Therefore, the presence of h-Na_3_Bi phase after the first sodiation is rather unusual as the presence of h-Na_3_Bi has been linked to long-term deactivation of the Bi metallates.^22,24^ Furthermore, this suggests that the Bi particles formed during the initial sodiation are slightly larger than those observed for other materials with similar chemistry. This indicates that the size and mobility of the alloying particles could be tuned by customizing the surrounding matrix.

To look for possible variations in the cycling mechanism at different rates, another *operando* measurement was performed with a current density of 0.1 A g^–1^ (Figure 1d–f). As expected, the measurements at 0.1 and 1 A g^–1^ showed the same general cycling mechanism. However, one significant difference was observed: at 1 A g^–1^, the diffraction peaks for the Na–Bi phases are less defined than those for the 0.1 A g^–1^ measurement. The challenges with performing a high-rate *operando* XRD measurement forced us to find an acceptable compromise between counting statistics and time resolution while maintaining the electrochemical performance, which resulted in less defined diffraction peaks. However, an alternative explanation arises from the lack of time required for the crystallization of the observed phases at high cycling rates, and, therefore, the corresponding peaks do not reach their maximum intensity and sharpness. The limited intensity of the diffraction peaks observed at the 1 A g^–1^ measurement made it difficult to separate the h-Na_3_Bi phase from c-Na_3_Bi and it was therefore not included in the surface Rietveld refinement.

In addition to the rate comparison, the measurement conducted at 0.1 A g^–1^ provided more detailed insights into the cycling mechanism. Because of the sharper and more intense diffraction peaks, it was easy to distinguish all the different Na_*x*_Bi phases and determine the exact moment of their formation. Phase fractions obtained from surface Rietveld refinement show that the c-Na_3_Bi phase forms before h-Na_3_Bi (Figure 1f). This is likely because the nucleation of Na_3_Bi within the NaBi particles results in the c-Na_3_Bi phase, a transformation shown to be more kinetically favorable than that from NaBi to the thermodynamically stable h-Na_3_Bi.^24^ The formation of h-Na_3_Bi starts later and proceeds at the cost of c-Na_3_Bi, indicating a transformation from the cubic to the hexagonal phase, possibly when the Na_3_Bi crystallites grow to a certain size. Another explanation is that a significant Na deficiency may stabilize in the c-Na_3_Bi structure and that h-Na_3_Bi forms as full sodiation is achieved: toward the end of sodiation, there is a significant timeslot after the disappearance of NaBi where only the two Na_3_Bi phases are present, while the electrochemistry is still active (gray areas in Figure 1f). This transformation cannot be treated as a relaxation process as it is electrochemically driven: ex situ XRD measurements of a sodiated sample conducted 2 weeks after cell disassembly confirmed the presence of c-Na_3_Bi and h-Na_3_Bi phases in an approximately 7:2 ratio (Figure S5).

Another notable observation is that the time that the NaBi phase is present is significantly shorter during desodiation than sodiation. This could be explained by the formation and dissolution of the SEI layer for each cycle as shown for Bi_2_MoO_6_ in our previous study.^22^ The formation of the SEI during sodiation consumes Na ions and, therefore, contributes to the capacity. This formation prolongs the duration of the sodiation, while the dissolution process during desodiation does not contribute significantly to the capacity. Another possibility is that the reaction paths during desodiation are slightly different from that for sodiation, which has previously been shown for the alloying reactions between Na and P.^30^

### Oxidation of Bi and the Nature of the Na–Fe–O Matrix

Most of the works on CAMs have been primarily focused on the electrochemically active materials, while the chemistry of the matrix and its interaction with the active component are still poorly understood. The XRD data described above combined with ex situ XAS measurements provided information on the local structure in the matrix material, the nature of its chemical interaction with the alloying particles, and how they influence each other.

In the desodiated state between ∼10 and 13 h (>1 V), the only detectable XRD peak from the working electrode is the peak corresponding to Bi metal (Figure 1d–f). However, a significant capacity (∼200 mAh g^–1^) is observed when assessing the contribution above 1 V during the first desodiation (Figure S4c), which suggests that additional chemical transformations other than (de)sodiation of Na_*x*_Bi particles take place at this voltage window. The ex situ XAS measurements of the Bi L3 edge confirmed that the oxidation state of Bi in the fully desodiated sample is higher than 0 (Figure S6). The FT EXAFS analysis also shows a clear peak corresponding to Bi–O bonds, together with Bi–Bi bonds similar to that observed for the Bimetal reference (Figure S6b,d). Thus, the capacity contribution above 1 V vs Na/Na^+^ is attributed to the oxidation of Bi, which was also shown by Surendran et al. through *operando* XAS measurements.^14^ This oxidation of Bi can be explained by Bi–O bonds at the interface between the Bi particles and the Na–Fe–O matrix. The contribution from the Bi–O bonds will be significant as long as the Bi particles are relatively small (<10 nm) so that the surface-to-volume ratio is large. In the region where Bi has a positive oxidation state, the main diffraction peak of Bi seems to shift slightly toward higher *Q* values during desodiation and back again during sodiation (Figure 1d). At this point, we are not able to explain this phenomenon.

There are no signs of the Na–Fe–O matrix in the *operando* XRD measurements as all of the peaks in the XRD pattern can be assigned to the alloying particles (Na_*x*_Bi phases) and the Na metal used as a counter electrode. The *operando* XAS study of Surendran et al. showed that Fe was redox active and contributed some to the capacity during the electrochemical cycling of BiFeO_3_.^14^ However, there are no clear plateaus in the (de)sodiation curves in our measurements that should correspond to the reduction or oxidation of Fe. Given the amorphous nature of the Fe-containing phase(s), it would likely exhibit a very gradual redox reaction that is difficult to observe in the (de)sodiation processes dominated by the electrochemistry of Bi. Our ex situ X-ray absorption near edge spectroscopy (XANES) measurements on cycled samples showed a shift in the edge position of Fe proving that it is electrochemically active (Figure S7), even though it is to a smaller extent than what Surendran et al. showed in their *operando* measurement.^14^ The apparent differences between Surendran’s and our results could originate from different morphologies of the particles and/or inherent differences between ex situ vs *operando* measurements. The XANES data also indicate a significant change in local coordination around the Fe atoms, where a pre-edge peak appears during the first sodiation (Figure S7a). This pre-edge feature is characteristic of tetrahedral coordination of Fe, similar to that in Fe_3_O_4_, as opposed to the octahedrally coordinated Fe in BiFeO_3_ that shows no significant pre-edge feature (Figure S7a). Apart from this small change in oxidation state and coordination of Fe, it is difficult to assess the Fe-containing phase(s). However, these results still suggest a dynamic nature of the Na–Fe–O matrix, which might be responsible for the degradation of the material.

### Material Degradation

**Figure 2:**
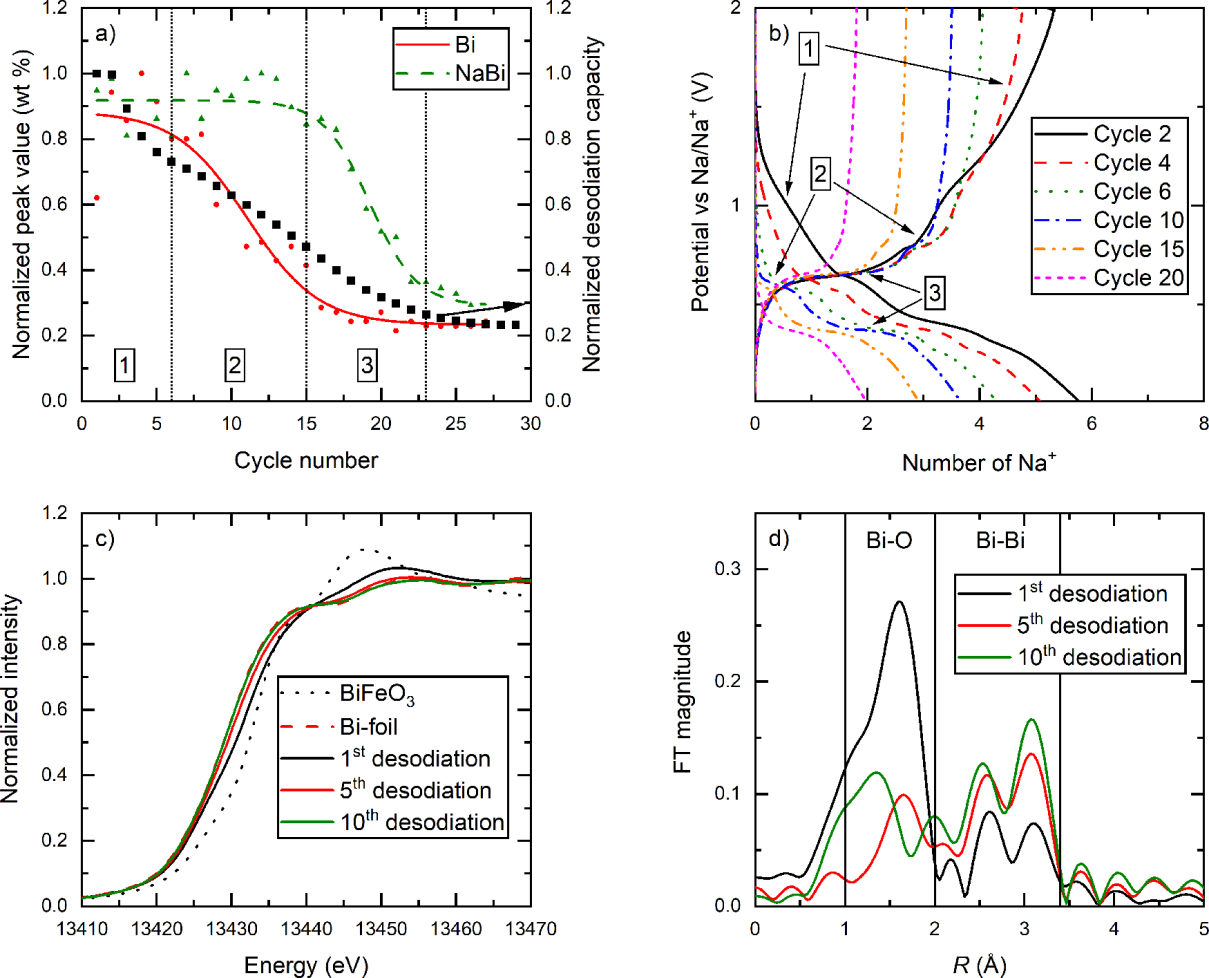
(a) Maximum normalized phase fractions for Bi and NaBi vs cycle number extracted from surface Rietveld refinement on *operando* XRD of BiFeO_3_ for the first 27 (de)sodiation cycles performed with a specific current of 0.2 A g^–1^ and a voltage range of 0.01–2.00 V vs Na/Na^+^. (b) Selected (de)sodiation curves plotted as potential vs Na/Na^+^ against the calculated number of Na^+^ ions transferred per formula unit obtained from the *operando* measurements shown in (a) highlighting the capacity decay between cycles 2 and 20. (c) XANES spectra of the Bi L3 edge for desodiated BiFeO_3_ at different stages of cycling compared to pristine BiFeO_3_ and Bi metal as references. (d) Corresponding FT EXAFS spectra showing the changes in Bi–O bonds.

To further understand the degradation processes in BiFeO_3_, we performed *operando* XRD for 27 cycles between 0.01 and 2.00 V in a half-cell configuration using Na foil as a counter electrode (Figure S8). Since BiFeO_3_ has the same cycling mechanism at different current densities, a current density of 0.2 A g^–1^ was selected to obtain a good compromise between total measurement time, time resolution, and data quality. Detailed analysis of the *operando* XRD data was combined with ex situ XAS measurements of the Bi L3 edge (Figures 2 and S9, SI). Based on the behavior of BiFeO_3_, the degradation mechanism could be divided into 3 well-defined regions, where each region is characterized by one specific redox reaction that determines the capacity decay.

Region 1 corresponds to the first 6 cycles and is assigned based on the disappearance of the oxidation of Bi (region 1 in Figure 2a,b). The XAS data from samples extracted after the first and fifth desodiation supports the statement that the capacity decay in this region is due to disappearance of Bi–O bonds at the interface between the Bi particles and the Na–Fe–O matrix (Figure 2c,d). All desodiated samples show characteristic signals for Bi–Bi bonds between 2 and 3.4 Å in the FT EXAFS graphs, indicating the presence of Bi metal (Figure 2d). At the same time, the position of the Bi XANES edge after first desodiation is between the position of Bi metal (Bi^0^) and BiFeO_3_ (Bi^3+^), meaning that Bi in this sample has a positive average oxidation state roughly between 1 and 2 (Figure 2c). Furthermore, there are clear signals in the FT EXAFS graphs of Bi–O bonds after the first desodiation between the *R* values of 1 and 2 Å, which mostly disappear after the fifth desodiation likely due to coalescence of Bi leading to larger particles inside the Na–Fe–O matrix. The analysis of the diffraction patterns in the fully desodiated state reveals that the diffraction peaks corresponding to the Na_*x*_Bi alloys become more pronounced as cycling progresses, confirming an increase of crystallite size (Figure S8, SI). This provides a rationale for the disappearance of the oxidation reaction as bigger particles will have a smaller surface-to-volume ratio and, therefore, fewer Bi–O bonds.

Between cycles 6 and 15 (region 2) the diffraction peaks of Bi gradually disappear together with the (de)sodiation plateaus corresponding to the Bi ⇋ NaBi reaction (Figure 2a,b). The same was observed for Bi_2_MoO_6_ between cycles 4 and 9 (Figure S10, SI).^22^ From cycles 15–26 for BiFeO_3_ (Figure 2a,b) and cycles 9–13 for Bi_2_MoO_6_ (Figure S10, SI), the intensities of the NaBi peaks are also greatly reduced, indicating that the NaBi ⇋ Na_3_Bi alloying reaction becomes partially irreversible and that the system locks itself in the sodiated state (region 3). Following this, the capacity stabilizes at ∼20% of its initial desodiation capacity, and the main capacity contribution comes from some redox activity between NaBi and Na_3_Bi.

**Figure 3:**
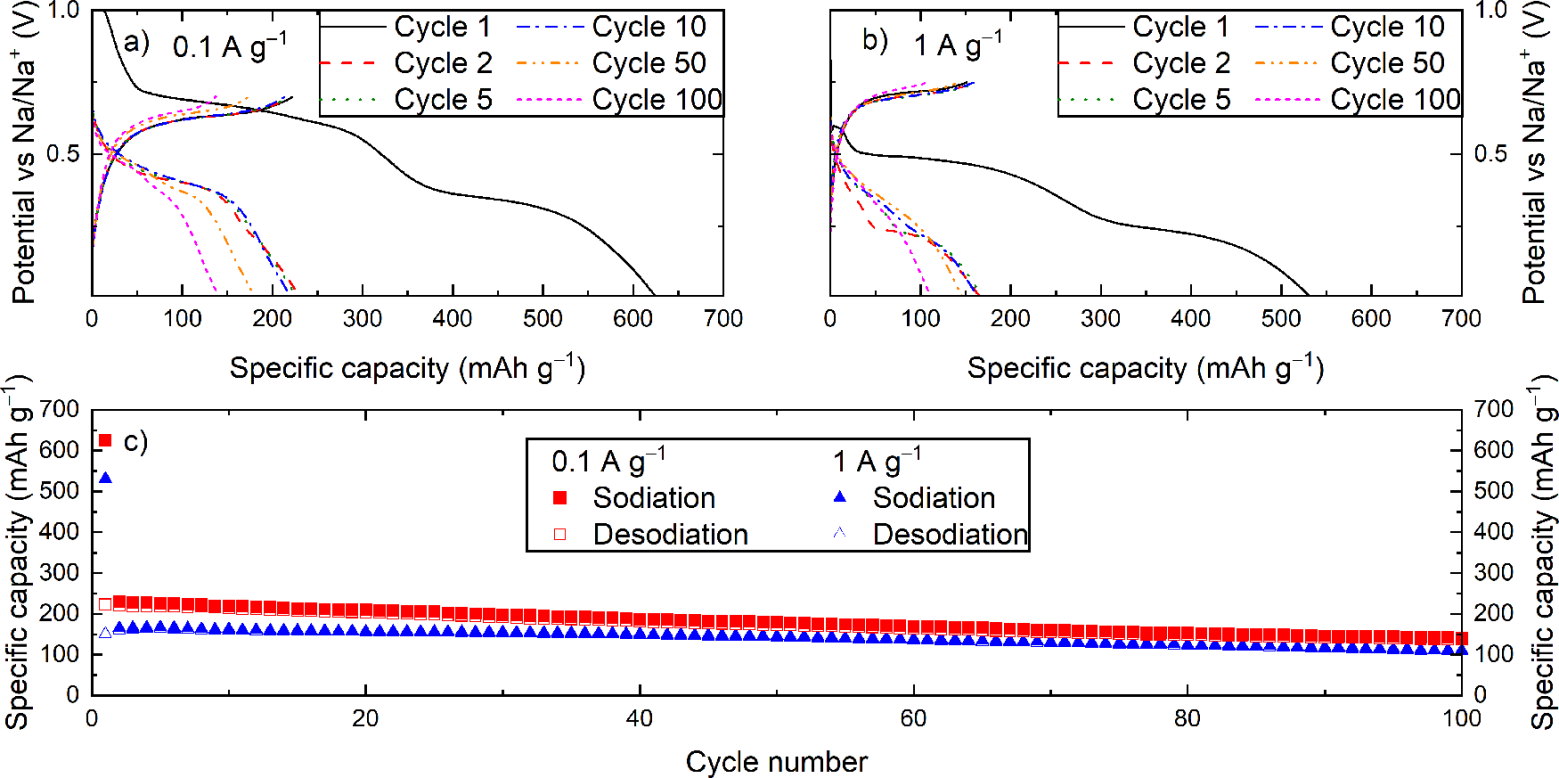
Selected (de)sodiation curves of BiFeO_3_ cycled at (a) 0.1 A g^–1^ with a voltage range of 0.01–0.70 V vs Na/Na^+^ and (b) 1 A g^–1^ with a voltage range of 0.01–0.75 V. (c) Specific (de)sodiation capacity per cycle derived from the measurements in (a) and (b).

Regions 1–3, described above, separate the capacity degradation according to the key chemical processes determining the capacity fading, with the main trigger being growth of the alloying particles inside the Na–Fe–O matrix. This increase in the particle size is most likely driven by the electrochemical sintering of the Na_*x*_Bi particles. In the pursuit of the origin of the capacity degradation, we reduced the upper cutoff voltage to 0.7 V vs Na/Na^+^ to cycle the material only between NaBi and Na_3_Bi using a current density of 0.1 A g^–1^ (Figure 3a,c). This new cutoff voltage limited the reversible capacity to a maximum of ∼230 mAh g^–1^ but increased the cycling stability significantly as the capacity was only reduced to ∼140 mAh g^–1^ after 100 cycles. The performance at a high rate (1 A g^–1^) was also well maintained (Figure 3b,c). This performance is still not sufficient to be of commercial interest; however, it demonstrates that the electrochemical isolation of the NaBi ⇋ Na_3_Bi reaction leads to stable cycling. Galvanostatic cycling up to 0.90 V vs Na/Na^+^ was also performed but showed similar capacity fading as during cycling to 2.00 V vs Na/Na^+^ (Figure S11). This showed that the Bi ⇋ NaBi reaction and the oxidation of Bi are the main contributors to capacity degradation.

The exact reason the capacity from the Bi ⇋ NaBi reaction and the surface oxidation of Bi decay so quickly is difficult to know with certainty, but it is likely that the large structural changes occurring during cycling are involved. The large volume expansion of ∼250% for the Bi ⇋ h-Na_3_Bi reaction could lead to particle cracking, which is potentially the main reason for the poor cycling stability of pure Bi metal.^24,31^ The Na–Fe–O matrix surrounding the Bi nanoparticles formed during the conversion reaction should mitigate some of these problems, but the growth of the alloying particles and the structural changes of the matrix including redox activity on Fe still lead to limited cycling stability. It may be that the matrix and the alloying particles are stable when cycling between NaBi and Na_3_Bi but that the transition between Bi and NaBi leads to significant movements in the matrix allowing the alloying particles to coalesce and induce capacity degradation. A more detailed understanding of the structural changes in the matrix and the growth of the Bi particles, especially above 0.7 V vs Na/Na^+^, could provide the knowledge needed to stabilize the full two-step (de)alloying reaction to reach an acceptable specific capacity. Finding a material that forms a stable matrix, which can keep the Bi particles in place while still allowing the alloying reactions to occur, will probably be the recipe for a CAM anode with good cycling stability.

## Conclusions

Using *operando* XRD, both at high rates and over 27 (de)sodiation cycles, we have revealed some new insights into the cycling mechanism of BiFeO_3_. The results reveal that the general cycling mechanism is the same at 1 and 0.1 A g^–1^. The first sodiation shows an irreversible conversion reaction, forming Bi nanoparticles embedded in a Na–Fe–O matrix. During the following cycles, the main contributor to the capacity is the two-staged alloying mechanisms going from Bi to NaBi and Na_3_Bi. We observe both the hexagonal and cubic versions of Na_3_Bi, where c-Na_3_Bi forms before h-Na_3_Bi during sodiation. There is also a significant capacity contribution above 1 V vs Na/Na^+^ during desodiation, attributed to the oxidation of the Bi nanoparticles, resulting in Bi–O bonds on the interface toward the Na–Fe–O matrix. This contribution gradually disappears during the first 6 cycles. The following capacity decay is explained by the increasing irreversibility of the alloying reactions where the equilibrium is shifted toward the sodiated phases. Especially for the Bi ⇋ NaBi reaction, which disappears completely. When reducing the upper cutoff voltage to isolate the NaBi ⇋ Na_3_Bi reaction, the cycling stability was significantly improved and the high-rate performance was maintained, showing that this reaction in itself is stable. There is still not much knowledge of the Na–Fe–O matrix, but ex situ XAS showed that Fe is electrochemically active and contributes a bit to the capacity. More detailed structural studies are needed to fully understand the nature of the Na–Fe–O matrix and the movement and growth of the Bi particles. This will hopefully enable us to design CAMs with good cycling stability, combined with the already established benefits of high capacity and good rate capabilities.

## Experimental Section

### Synthesis

BiFeO_3_ was synthesized by a sol–gel method based on the study of Ma et al.,^32^ where 1.2 × 10^–2^ mol (5.822 g) Bi(NO_3_) ×5H_2_O (98%, Sigma-Aldrich) and 1.0 × 10^–2^ mol (4.040 g) Fe(NO_3_)_3_ × 9H_2_O (≥98%, Sigma-Aldrich) were mixed with 8–10 mL of ethylene glycol (99.8%, Sigma-Aldrich) under magnetic stirring at 100 rpm and 60 °C for 1 h. Subsequently, the temperature was increased to 100 °C to allow for a slow evaporation of the solvent via autocombustion. The resulting dry gel was then transferred to an Al_2_O_3_ crucible and calcined in air at 350 °C for 3 h in a muffle oven (Carbolite Furnaces CWF 1200) and cooled to room temperature. The product was then crushed to powder in an agate mortar, pressed to a pellet, and treated at 650 °C for 8 h. To decrease the particle size, 2 g of the obtained product was ball-milled with 85 g of 3 mm stainless steel balls in a Pulverisette 7 Premium Line (Fritsch) for 20 min at 250 rpm in an argon atmosphere.

### Material Characterization

The XRD samples were prepared by mixing the obtained product with isopropanol (≥99.7%, VWR) and dispersing it on a flat glass plate mounted on the sample holder. All XRD patterns of uncycled samples were collected using a D8 Discover diffractometer (Bruker, Cu-source, λ = 1.5406 Å) with a point detector and fluorescence correction in the 2θ range 10–100° (*Q* = 0.71–6.25 Å^–1^). One scan lasted ∼3 h. The morphology of the BiFeO_3_ particles was investigated with a high-resolution Hitachi SU8230 cold-field emission scanning electron microscope operated with an acceleration voltage of 5 kV. The images were generated by secondary electrons, with a working distance of 8.8 mm and magnifications ranging from 1000 to 25,000. The powder was spread out on carbon tape, attached to the sample holder, to fix the powder and ensure good conductivity.

### Electrode Preparation

The battery electrodes were made by combining the active material (BiFeO_3_), binder (carboxymethylcellulose sodium salt, CMC, Sigma-Aldrich), conductive agent (Carbon black, Super P, Timcal), and solvent (distilled H_2_O) in an 8:1:1 ratio into a slurry in a Thinky mixer (ARE 250). The mixing procedure consisted of 5 min of mixing at a speed of 2000 rpm, followed by a defoaming step for 1.5 min at 750 rpm. After the mixing procedure, which was repeated if necessary, the slurry was coated onto a dendritic Cu foil (Schlenk, 10 μm thick) with a coating height of 300 μm. The resulting electrode sheets were dried in air overnight, cut into 15 mm discs the following day, and were further dried in a Büchi oven at 60 °C under dynamic vacuum for 3 h. Afterward, the electrodes were placed into a glovebox (MBraun Labmaster, H_2_O and O_2_ < 0.1 ppm) for coin cell assembly.

### Electrochemical Characterization

Electrochemical characterization was performed in half-cells with metallic Na as the counter electrode. Na chunks (Sigma-Aldrich) were cut into smaller pieces, rolled flat, and then stamped into 14 mm discs. The discs were then brushed on each side with a toothbrush (medium, First Price) to ensure a fresh surface. Each Na disc was placed in a coin cell (CR2032, stainless steel, 304, Pi-Kem) together with a separator (Whatman GE, 16 mm) and wetted with 80 μL of electrolyte and the working electrode before the battery was closed using an automatic coin cell crimper (Hohsen). The electrolyte was mixed inside the glovebox and consisted of 1 M NaPF_6_ (Fluorochem) in propylene carbonate (PC, 99.7%, Sigma-Aldrich) with 5% fluoroethylene carbonate (FEC, 99%, Sigma-Aldrich) as an additive.

Galvanostatic cycling (GC) of the batteries was carried out with a Neware battery tester (CT-4008T-5V10mA-164) at 25 °C with a lower cutoff voltage of 0.01 V and current densities of 0.1 and 1 A g^–1^ with respect to the mass of BiFeO_3_. The active mass loading of the electrodes was between 1 and 2 mg cm^–2^. The upper cutoff voltage was 2 V for most of the measurements, but it was reduced to 0.7 V for one measurement at 0.1 A g^–1^ and to 0.75 at 1 A g^–1^ in order to isolate the NaBi ⇋ Na_3_Bi reaction. The higher upper cutoff voltage for the 1 A g^–1^ measurement was chosen to account for the increased overpotential at higher current densities. Cyclic voltammetry (CV) measurements were performed with an MPG2 battery cycler (BioLogic) using a voltage window of 0.01–2.00 V and sweep rates of 0.1 and 1 mV s^–1^.

### *Operando* XRD

Laboratory manufactured *operando* cells similar to the one presented by Drozhzhin et al. with glassy carbon windows were used for the *operando* XRD measurements.^33^ The electrode for the *operando* measurement over the course of 2 (de)sodiation cycles at 0.1 A g^–1^ was made by drop-casting the slurry directly onto one of the cell halves to optimize the XRD signals. The measurement over 27 cycles used normal electrodes on Cu foil to ensure good electrochemical performance. Apart from this, the assembly was performed as similar as possible to the normal coin cells. The GC measurements of these two *operando* measurements were performed using an SP150 battery cycler (BioLogic) with a voltage range of 0.01–2.00 V and current densities of 0.1 A g^–1^ (2 cycles) and 0.2 A g^–1^ (27 cycles). The XRD measurements were performed simultaneously with continuous still scans (10 min scan^–1^, *Q* = 1.26–3.65 Å^–1^) using a Dectris Eiger2 R 500 K detector on a Bruker D8 Advance diffractometer (Mo source, λ_kα1_ = 0.7093 Å, λ_kα2_ = 0.7136 Å, focusing mirror primary optic).

The high-rate *operando* XRD measurement with a current density of 1 A g^–1^ and a voltage range of 0.01–2.00 V was also performed over the course of 2 cycles. This measurement was performed with synchrotron radiation at BM31, which is a part of the Swiss-Norwegian Beamlines (SNBL), at ESRF with a wavelength of 0.24486 Å and a Pilatus CdTe 2 M detector from DECTRIS. Each scan had an exposure time of 20 s with 15 repetitions that was averaged, giving a total measurement time of 5 min per data point. The electrode used for this measurement was coated with a wet thickness of 500 μm on a Cu foil.

### Ex Situ XAS

Electrodes for ex situ XAS were prepared in the same way as that for normal coin cells. The batteries were cycled at 0.1 A g^–1^ between 0.01 and 2.00 V until the desired stage of cycling (specified in main text) where they then went through a constant voltage step where the cutoff voltage was held until the current was below 20 μA. The coin cells were then transferred to the glovebox and disassembled with a coin cell disassembling tool (Hoshen). The electrodes were carefully extracted from the disassembled coin cells, cleaned with ∼0.5 mL of DEC per electrode, and dried for 1 h inside the glovebox. Following this, the electrode material was scraped from the Cu foil, ground carefully in a mortar, and filled in 1 mm borosilicate glass capillaries that were sealed with UV glue (Bondic). The XAS measurements on the Bi L3 edge were performed in transmission mode with ion chamber detectors in the energy range of 13.32–14.10 keV, 0.7 eV step size, and 200 ms exposure giving a measurement time of 3 min and 44 s per scan. Each sample was measured with 2 repetitions, which were averaged in Athena.^34^ The Fe K edge was measured in fluorescence mode with an energy range of 7.05–7.26 keV, 0.3 eV step size, and 200 ms exposure, leading to a total measurement time of 2 min and 20 s.

### Software and Data Treatment

To visualize the crystal structure of BiFeO_3_, we utilized Vesta.^35,36^ J-edit and Topas v6^37^ were used for Rietveld refinements, while EC-lab and software from Neware were used to process electrochemistry data. Python 3 and Origin 2020 were utilized for further processing and plotting of the data, including python scripts provided by the BM31 staff. The Athena software was used for processing and analyzing the XAS data.^34^

## Data Availability

All the background data for this publication including procedures and scripts for data treatment are available at dataverse.no.^38^ Additional data and analysis from the BiFeO_3_ system are also available.^39^

## Supporting Information Available

XRD and SEM data of pristine BiFeO_3_; (de)sodiation curves and cycling voltammograms at different cycling conditions; ex situ XRD and XAS data from cycled BiFeO_3_ samples; contour plot from *operando* XRD of BiFeO_3_ over 27 cycles with corresponding dQ/dV curves; and results from surface Rietveld refinement of *operando* XRD of Bi_2_MoO_6_ (PDF)

## ABBREVIATIONS

CAM: conversion-alloying material
CMC: carboxymethylcellulose sodium salt
CV: cyclic voltammetry
ESRF: European Synchrotron Radiation Facility
EXAFS: extended X-ray absorption fine structure
FEC: fluoroethylene carbonate
FT: Fourier transform
GC: galvanostatic cycling
LIB: Li-ion battery
NIB: Na-ion battery
NFR: Norges Forskningsråd (The Research Council of Norway)
PC: propylene carbonate
RECX: Resource Centre for X-ray Diffraction and Scattering
SEM: scanning electron microscopy
SEI: solid electrolyte interface
SNBL: Swiss-Norwegian beamlines
TSCT: total scattering computed tomography
TEM: transition electron microscopy
TM: transition metal
XANES: X-ray absorption near edge spectroscopy
XAS: X-ray absorption spectroscopy
XRD: X-ray diffraction
