# Supplementary material for: Unraveling the (De)sodiation Mechanisms of BiFeO3 at a High Rate with Operando XRD
Source: ACS Appl Mater Interfaces. 2024 Feb 27;16(10):12428–36. doi: 10.1021/acsami.3c17296 (PMC10941182; doi:10.1021/acsami.3c17296)
Supplement: Supplementary file 1 — am3c17296_si_001.pdf [file am3c17296_si_001.pdf]

Supporting Information: "Unravelling the (De)sodiation Mechanisms of $BiFeO_3$ at a High Rate with *Operando* XRD"

Anders Brennhagen[1*], Casper Skautvedt[1], Carmen Cavallo[2], David S. Wragg[1,3], Alexey Y. Koposov[1,3], Anja O. Sjåstad[1], Helmer Fjellvåg[1*].

[1]Centre for Materials Science and Nanotechnology, Department of Chemistry, University of Oslo, PO Box 1033, Blindern, N-0315, Oslo, Norway

[2]CENATE, Centrifugal Nanotechnology, Rakkestadveien 1, 1814, Askim, Norway

[3]Department of Battery Technology, Institute for Energy Technology (IFE), Instituttveien 18, 2007, Kjeller, Norway

E-mail: anders.brennhagen@smn.uio.no and helmer.fjellvag@kjemi.uio.no

Section S1: Structural and Morphological Characterization

$BiFeO_3$ takes a distorted perovskite structure described in space group R3c using a hexagonal unit cell (Figure S1b, Table S1). Due to the inert lone pair of the 6s orbitals of Bi^{3+} the FeO_6 and the BiO_{12} polyhedra are significantly distorted compared to the ideal perovskite structure. The secondary particles of the synthesized $BiFeO_3$ had a wide distribution in particle sizes between 1−500 µm in diameter, as shown by scanning electron microscopy (SEM), and they consisted of primary particles with sizes ranging from 100−500 nm (Figure S1c and d). To improve the electrochemical performance of the material, the sample was ball milled for 20 min at 250 rpm to pulverize the largest secondary particles without drastically changing the nanostructures of the material. The SEM images of the ball milled sample showed that the largest secondary particles after ball milling were <20 µm in diameter, while the primary particles were of the same size as the pristine sample (Figure S1e and f). The XRD pattern of the ball-milled sample was similar to the pristine sample (Figure S1a). The only significant difference was some peak broadening, which is probably due to strain from the ball milling.

Table S1: Structural information of the phases used for XRD analysis

Phase	Space group	Z	Unit cell parameters (Å)	Atomic sites					Source
				Atom	Wyckoff	x	y	z	
$BiFeO_3$	R3c:H (161)	4	a = 5.57893<br>c = 13.86953	Bi1<br>Fe1<br>O1	6a<br>6a<br>18b	0<br>0<br>0.444	0<br>0<br>0.018	0<br>0.2206<br>0.9525	ICSD: 192511
Bi_2O_3	$P2_1/c$ (14)	4	a = 5.8486<br>b = 8.1661<br>c = 7.5097	Bi1<br>Bi2<br>O1<br>O2<br>O3	4e<br>4e<br>4e<br>4e<br>4e	0.5240<br>0.0409<br>0.7800<br>0.2420<br>0.2710	0.1831<br>0.0425<br>0.3000<br>0.0440<br>0.0240	0.3613<br>0.7762<br>0.7100<br>0.1340<br>0.5130	COD: 9012546
Bi	R-3m (166)	6	a = 4.546<br>c = 11.862	Bi1	2g	0	0	0.23389	COD: 2310889
NaBi	P4/mmm (123)	1	a = 3.46<br>c = 4.8	Bi1<br>Na1	1a<br>1d	0<br>1/2	0<br>1/2	0<br>1/2	ICSD: 58816
h-Na_3Bi	$P6_3/mmc$ (194)	2	a = 5.448<br>c = 9.655	Bi1<br>Na1<br>Na2	2c<br>2b<br>4f	1/3<br>0<br>1/3	2/3<br>0<br>2/3	1/4<br>1/4<br>0.583	COD: 1010291
c-Na_3Bi	Fm-3m (225)	4	a = 7.66526	Bi1<br>Na1<br>Na2	4a<br>4b<br>8c	0<br>1/2<br>1/4	0<br>1/2<br>1/4	0<br>1/2<br>1/4	[1]

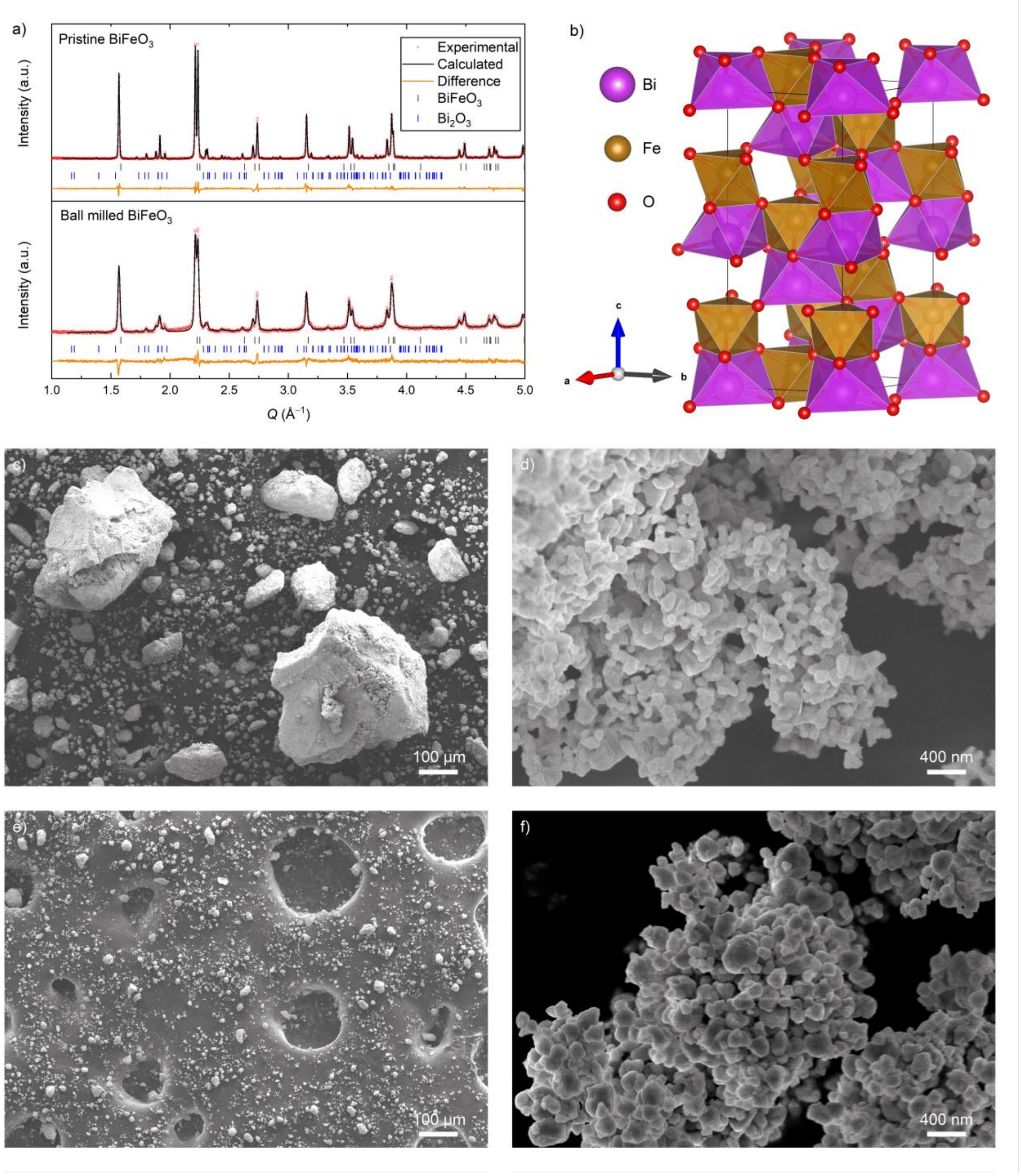

Figure S1: a) XRD with Rietveld refinement of pristine $BiFeO_3$ and the sample after ball milling for 20 min at 250 rpm. b) Visualization of the crystal structure of $BiFeO_3$ (space group: R3c). SEM images of c) pristine $BiFeO_3$ particles with magnification of 100 x, d) pristine $BiFeO_3$ at 25 000 x, e) ball milled $BiFeO_3$ at 100 x, f) ball milled $BiFeO_3$ at 25 000 x. All SEM images are generated by secondary electrons.

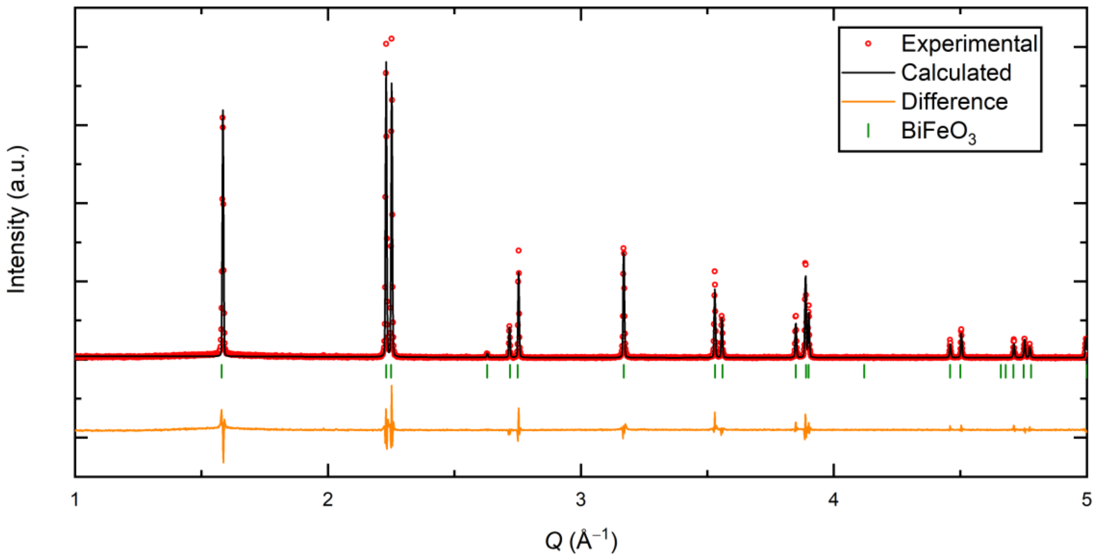

Figure S2: XRD pattern fitted with Rietveld refinement of pure $BiFeO_3$ obtained by sol-gel synthesis followed by leaching with an aqueous solution of 10% HNO_3.

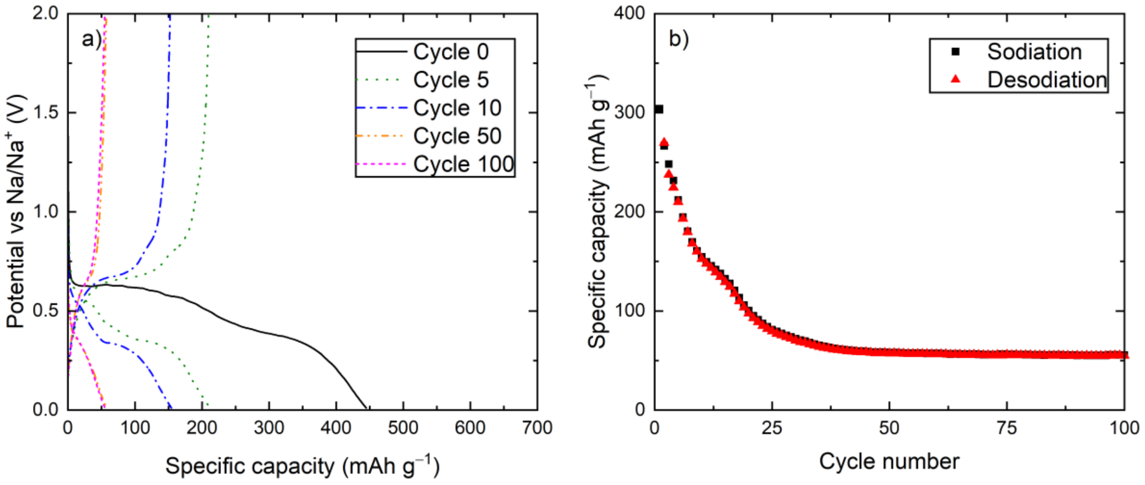

Figure S3: Galvanostatic cycling of leached $BiFeO_3$. a) (De)sodiation curves plotted as voltage vs specific capacity and b) specific capacity per cycle plot.

Section S2: Electrochemical Characterization

To evaluate the electrochemical performance of ball-milled $BiFeO_3$ in NIBs, we conducted several cyclic voltammetry (CV) and galvanostatic cycling (GC) measurements in half cells vs Na/Na^+. CV measurements with a sweep rate of 0.1 mV s^{-1} showed two distinct peaks during sodiation at ~0.6 and ~0.3 V (Figure S4a). The peak at ~0.6 V is larger during the first sodiation than in the following cycles, and it has a clear shoulder on the right side starting around 1 V. This peak (and shoulder) should correspond to the conversion reaction from $BiFeO_3$ to Bi and the first step of the alloying reaction forming NaBi (reaction (1)). In addition, some solid electrolyte interface (SEI) formation likely occurs in this region. The second peak is characteristic of the second alloying step where NaBi transforms into Na_3Bi (reaction (2)).

$$Na + Bi \leftrightharpoons NaBi \qquad (1)$$

$$2Na + NaBi \leftrightharpoons Na_3Bi \qquad (2)$$

During desodiation, the peaks corresponding to the dealloying reaction of Na_3Bi to NaBi is present at ~0.7 V and the peak at ~0.8 V corresponds to the formation of Bi (Figure S4a). In addition to these two peaks, there is a bump between 1.00 and 1.25 V that is related to the further oxidation of Bi [2]. At the higher sweep rate of 1 mV s^{-1}, the peaks are broader and shifted towards lower voltages during sodiation and higher voltages for desodiation compared to the measurement at 0.1 mV s^{-1} (Figure S4b). This is most pronounced during the first sodiation where the curve peaks around 0.1 V (shifted with 0.5 V), indicating that the initial conversion reaction has limited kinetics. The peaks corresponding to the alloying reactions during the following cycles are not shifted as much (roughly 0.1 V), indicating a lower overpotential and better kinetics.

To further evaluate the electrochemical performance of $BiFeO_3$ at different rates, GC measurements at 0.1 and 1 A g^{-1} were performed (Figure S4c and d). The initial sodiation capacity of $BiFeO_3$ was ~600 mAh g^{-1} at 0.1 A g^{-1}. The measured capacity is higher than the theoretical capacity of the conversion and alloying to Na_3Bi and a Na–Fe–O matrix with Fe^{3+}, which is 514 mAh g^{-1}. Some of this deviation between theoretical and experimental capacity could be explained by redox activity of Fe, which is known to happen for $BiFeO_3$ [2]. The rest of the deviation probably comes from SEI formation, sodiation of the conductive carbon and uncertainty from weighing of the electrodes. The contribution from the conductive carbon is estimated to be ~15 mAh g^{-1} and the uncertainty of the weighing is ~10% for the electrodes used in this study [3]. The most pronounced difference at the different cycling rates is the conversion reaction during the first sodiation where the reaction starts at ~1.0 V in the 0.1 A g^{-1} measurement compared to ~0.5 V at 1 A g^{-1}. This is consistent with the CV measurements (Figure S4a and b), which also showed a significant difference in the first sodiation. Apart from this, the measurements at the two current densities are very similar, showing that the material can handle high current densities without compromising on performance. The cycling stability of $BiFeO_3$ is challenging as the capacity drops from ~450 mAh g^{-1} at the second cycle to approximately ~130 mAh g^{-1} after 25 cycles (Figure S4e). Following this, the capacity is reasonably stable up to at least 100 cycles, where the capacity ends up at ~120 mAh g^{-1} and ~70 mAh g^{-1} for 0.1 A g^{-1} and 1 A g^{-1}, respectively (Figure S4f). We have ~12 wt% Bi_2O_3 in our sample, which is known to be electrochemically active in NIBs and show similar performance as $BiFeO_3$ [4-6]. The Bi_2O_3 impurities introduces some uncertainties to the specific capacities presented in this paper, but should not alter the key findings.

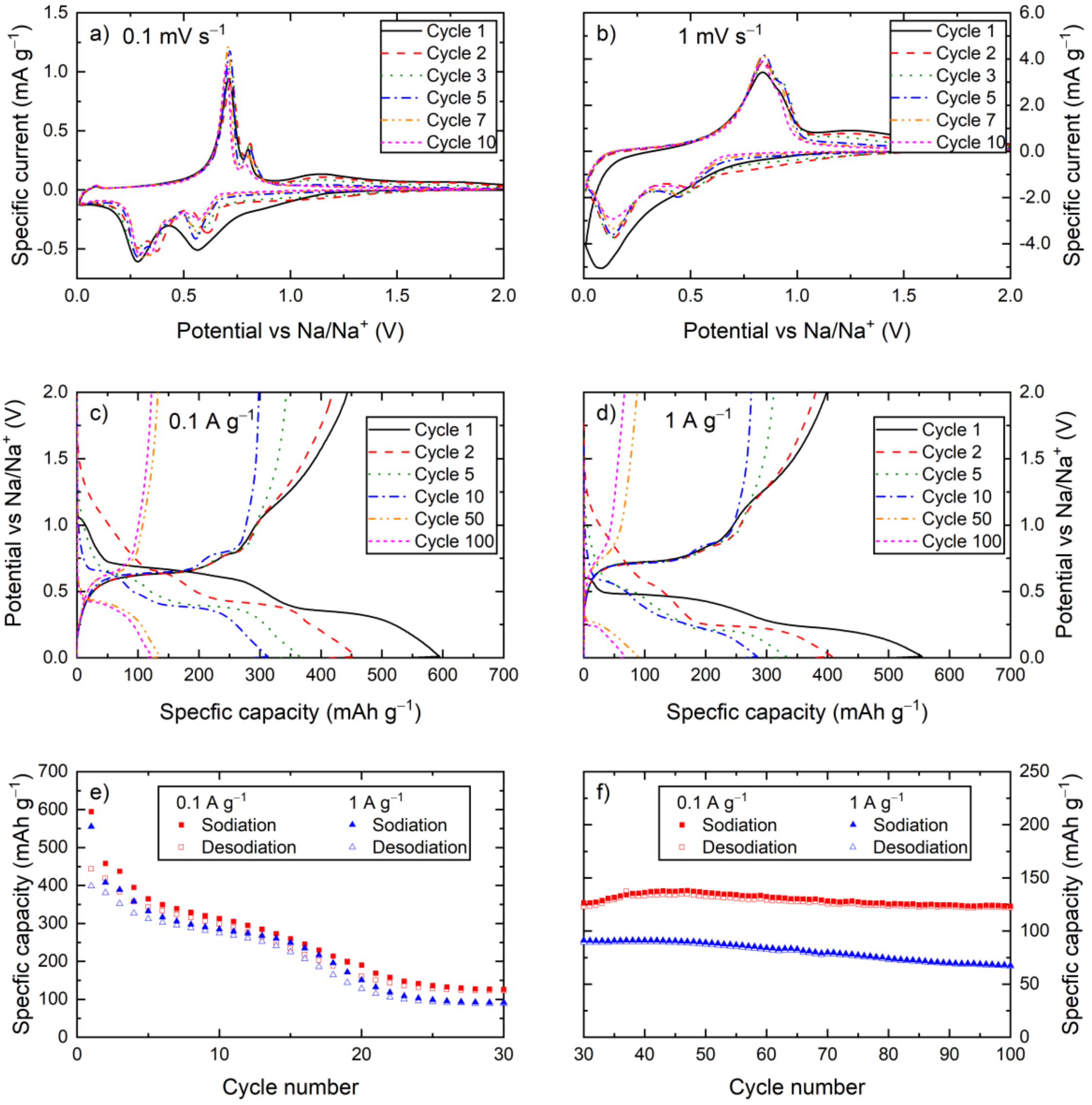

Figure S4: Electrochemical characterization of ball-milled $BiFeO_3$. a) and b) cyclic voltammograms obtained from CV measurements with a voltage range of 0.01−2.00 V vs Na/Na^+ and sweep rates of 0.1 mV s^{-1} and 1 mV s^{-1}, respectively. c) and d) (de)sodiation curves of selected cycles derived from GC measurements with current densities of 0.1 A g^{-1} and 1 A g^{-1}, respectively. e) and f) capacity per cycle plot comparing the cycling behavior at the different current rates, derived from the GC measurements.

Section S3: Other Supporting Figures

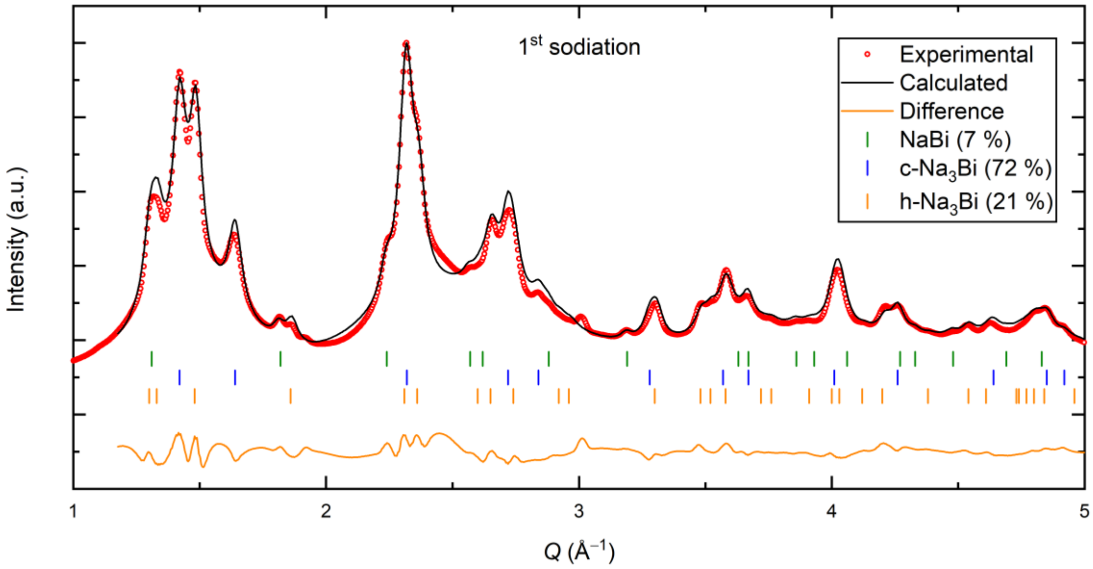

Figure S5: Ex situ XRD measured on a sealed capillary with $BiFeO_3$ extracted after the 1st sodiation. The sample was extracted from 5 coin cells, cycled at 0.1 A g^{-1} to 0.01 V, to obtain enough powder. Rietveld refinement with corresponding phase fractions in wt % provided in the legend.

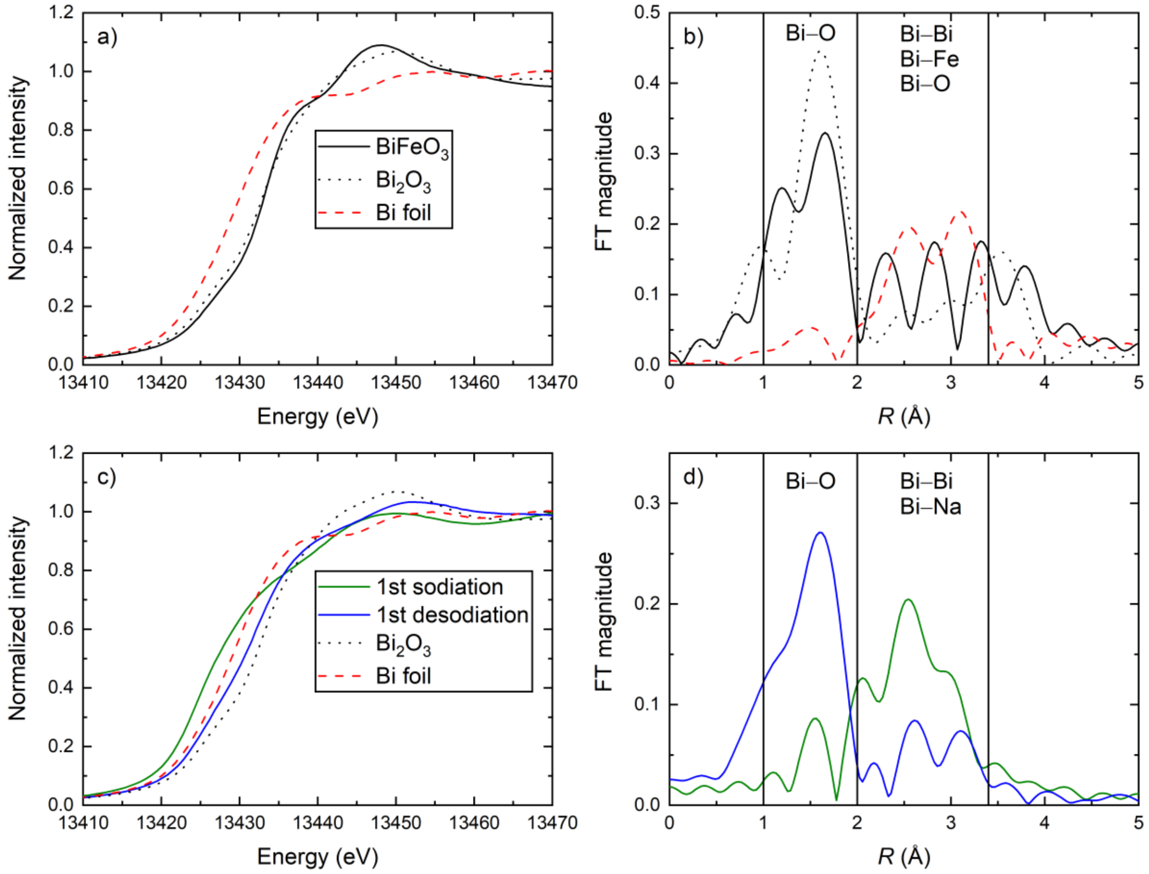

Figure S6: XAS measurements of the Bi L3 edge. a) XANES and b) FT EXAFS spectra of $BiFeO_3$, Bi_2O_3 and Bi-foil. c) XANES spectra of $BiFeO_3$ samples after 1st sodiation and desodiation compared to Bi_2O_3 and Bi-metal foil as references. d) Corresponding FT EXAFS spectra of 1st sodiation and desodiation.

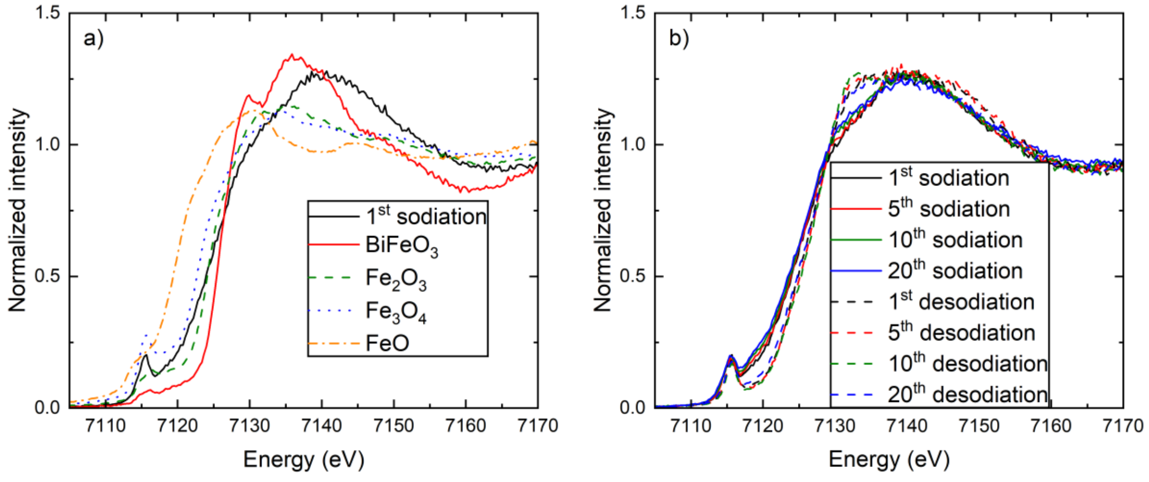

Figure S7: XANES spectra of the Fe K edge. a) Pristine $BiFeO_3$ and $BiFeO_3$ after 1st sodiation compared to Fe-oxide references and b) cycled $BiFeO_3$ samples at different stages of cycling.

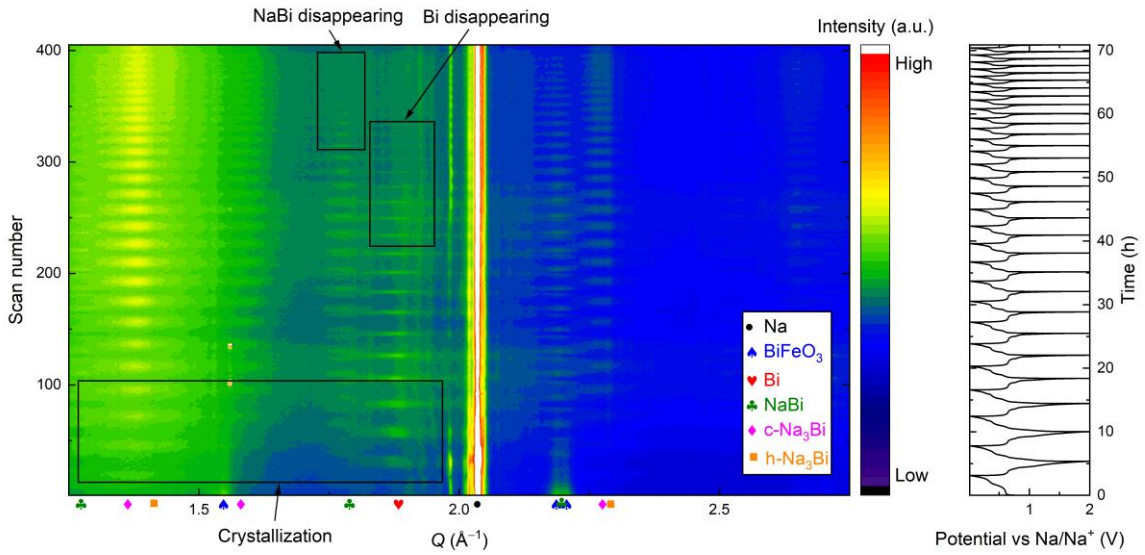

Figure S8: ***Operando*** **XRD visualized as a contour plot (left) with corresponding (de)sodiation curves (right) derived from a measurement of $BiFeO_3$ over the course of 27 cycles with current density of 0.2 A g^{-1} and voltage range of 0.01−2.00 V vs Na/Na^+.**

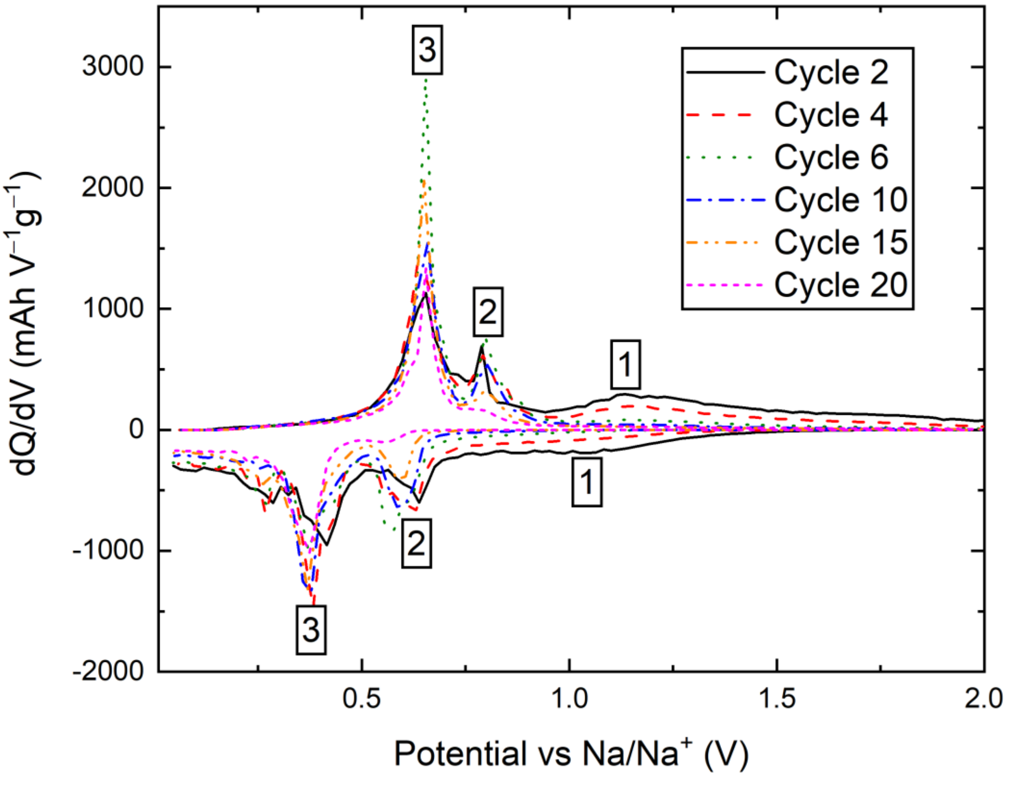

Figure S9: dQ/dV plot derived from (de)sodiation curves presented in Figure 2b in the main article.

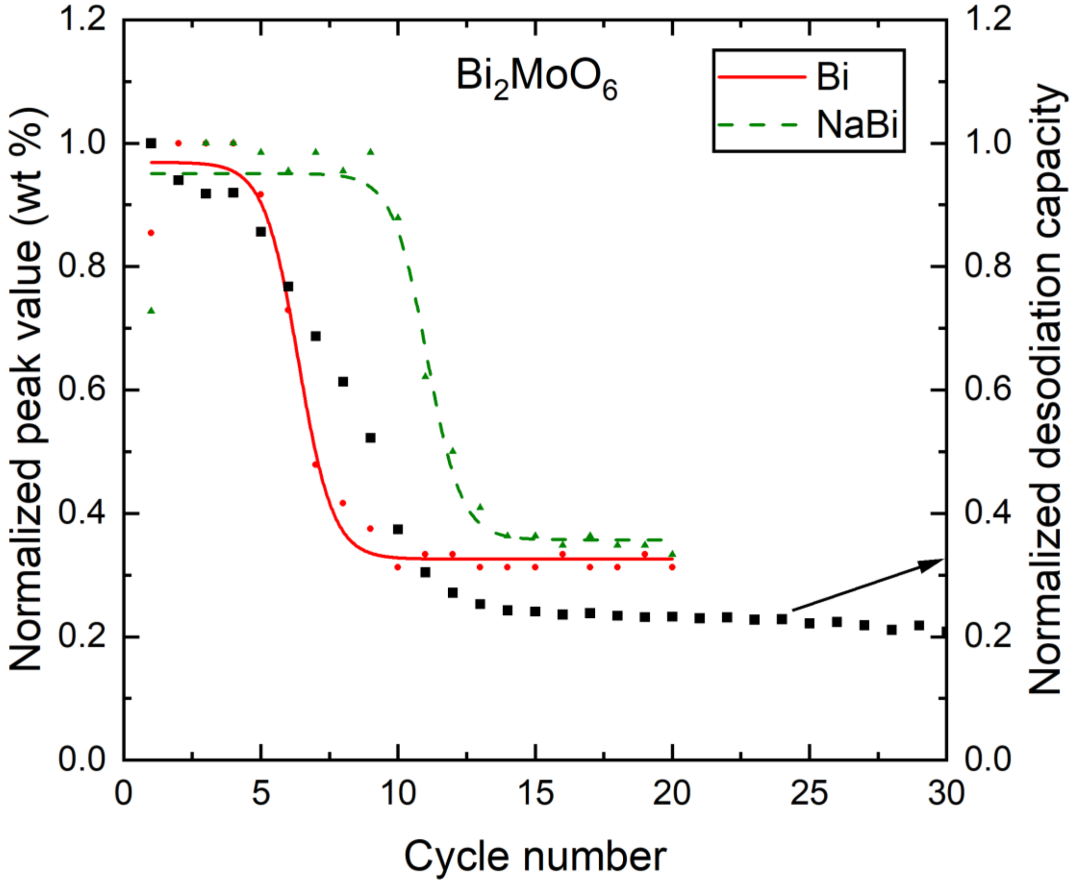

Figure S10: Maximum normalized phase fractions for Bi and NaBi vs cycle number extracted from surface Rietveld refinement on *operando* XRD measurement performed with a specific current of 0.1 A g^{-1} and a voltage range of 0.01−2.00 V vs Na/Na$^+$ from our previous study on Bi$_2$MoO$_6$ [3].

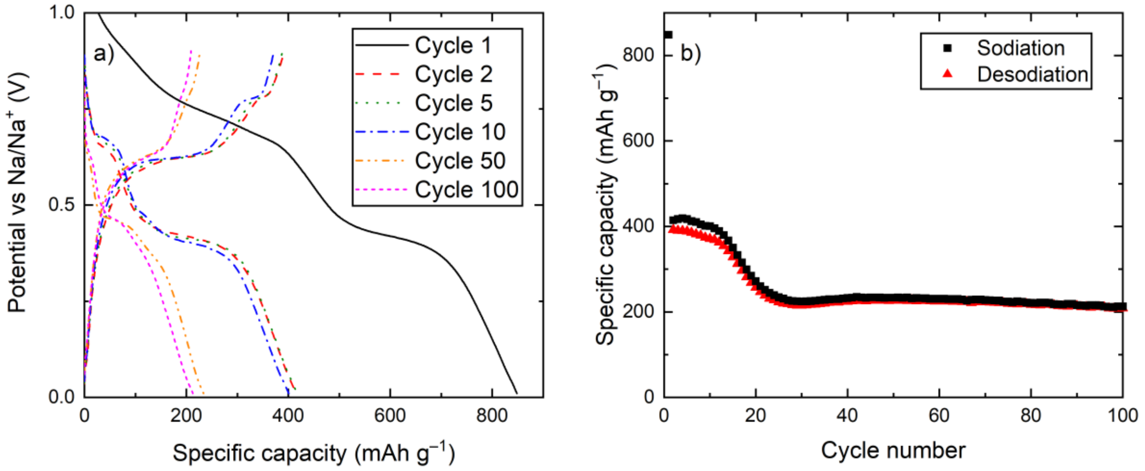

Figure S11: a) (De)sodiation curves and b) capacity per cycle plot from GC measurement on BiFeO$_3$ cycled between 0.01–0.90 V with a current density of 0.1 A g^{-1}.

References

[1] Sottmann, J.; Herrmann, M.; Vajeeston, P.; Hu, Y.; Ruud, A.; Drathen, C.; Emerich, H.; Fjellvag, H.; Wragg, D. S. How Crystallite Size Controls the Reaction Path in Nonaqueous Metal Ion Batteries: The Example of Sodium Bismuth Alloying. *Chem. Mater.* **2016**, *28(8)*, 2750-2756.

[2] Surendran, A.; Enale, H.; Thottungal, A.; Sarapulova, A.; Knapp, M.; Nishanthi, S.; Dixon, D.; Bhaskar, A. Unveiling the Electrochemical Mechanism of High-Capacity Negative Electrode Model-System $BiFeO_3$ in Sodium-Ion Batteries: An in Operando XAS Investigation. *ACS Appl. Mater. Interfaces* **2022**, *14(6)*, 7856-7868.

[3] Brennhagen, A.; Cavallo, C.; Wragg, D. S.; Vajeeston, P.; Sjåstad, A. O.; Koposov, A. Y.; Fjellvåg, H. Operando XRD Studies on Bi_2MoO_6 as Anode Material for Na-Ion Batteries. *Nanotechnology* **2022**, *33(18)*, 185402.

[4] Mei, J.; Liao, T.; Ayoko, G. A.; Sun, Z. Two-Dimensional Bismuth Oxide Heterostructured Nanosheets for Lithium-and Sodium-Ion Storages. *ACS Appl. Mater. Interfaces* **2019**, *11(31)*, 28205-28212.

[5] Kim, M.-K.; Yu, S.-H.; Jin, A.; Kim, J.; Ko, I.-H.; Lee, K.-S.; Mun, J.; Sung, Y.-E. Bismuth Oxide as a High Capacity Anode Material for Sodium-Ion Batteries. *Chem. Commun.* **2016**, *52(79)*, 11775-11778.

[6] Sun, J.; Li, M.; Oh, J. A. S.; Zeng, K.; Lu, L. Recent Advances of Bismuth Based Anode Materials for Sodium-Ion Batteries. *Mater. Technol.* **2018**, *33(8)*, 563-573.
